# Rationalism, Empiricism, and Evidence-Based Medicine: A Call for a New Galenic Synthesis

**DOI:** 10.3390/medicines5020040

**Published:** 2018-04-25

**Authors:** William M. Webb

**Affiliations:** Medical Scientist Training Program, University of Alabama at Birmingham, Birmingham, AL 35294, USA; wmwebb89@uab.edu

**Keywords:** epistemology, evidence-based medicine, medical education, rationalism, empiricism, Galen

## Abstract

Thirty years after the rise of the evidence-based medicine (EBM) movement, formal training in philosophy remains poorly represented among medical students and their educators. In this paper, I argue that EBM’s reception in this context has resulted in a privileging of empiricism over rationalism in clinical reasoning with unintended consequences for medical practice. After a limited review of the history of medical epistemology, I argue that a solution to this problem can be found in the method of the 2nd-century Roman physician Galen, who brought empiricism and rationalism together in a synthesis anticipating the scientific method. Next, I review several of the problems that have been identified as resulting from a staunch commitment to empiricism in medical practice. Finally, I conclude that greater epistemological awareness in the medical community would precipitate a Galenic shift toward a more epistemically balanced, scientific approach to clinical research.

## 1. Introduction

Despite signs that interest in medical humanities programs is increasing among prospective medical students [1,2], a major enrollment gap exists between the humanities and the hard sciences in pre-medical and undergraduate medical education. According to data released by the American Association of Medical Colleges, humanities majors comprise less than 4% of all applicants to medical schools—philosophy and history majors necessarily less [3]. Despite moves by the MCAT^®^ Exam to incorporate more humanistic criteria in its content and evaluation starting in 2015, no medical school in the United States requires coursework in philosophy from its applicants. Perhaps nowhere have the consequences of this gap been more keenly felt than in the rise and reception of the evidence-based medicine (EBM) movement and the use of expert advice in medical decision making.

Beginning in the 1990s, EBM emerged as a “new paradigm” for teaching clinical medicine that emphasized decision-making based on empirical evidence from controlled, randomized studies. The expectation was that such a paradigm would replace anecdotal experience, tradition, and—interestingly, from an epistemological perspective—reliance on deductive reasoning from mechanistic theories rooted in the basic sciences [4]. In this article, I argue that a widespread lack of training in philosophy and the history of medicine has resulted in the application of EBM-based principles and approaches in ways that privilege empiricism over rationalism. Taking the position that insights from both of these epistemological traditions are necessary in medical practice, I argue that proponents of EBM should pursue a synthesis of rationalism and empiricism and thereby adopt a more epistemically balanced and historically conscious approach.

The longstanding tension between rationalism and empiricism in medical epistemology has ancient origins reaching back at least as far as the writings of Galen, the celebrated 2nd-century CE Roman physician, and it continues well into the 21st century. Therefore, it is not my intention in this article to provide a thorough account of epistemology in medical science. Rather, it is to show that inasmuch as the EBM phenomenon represents a swinging of the pendulum toward empiricism, it runs the risk of being applied outside of its proper epistemic boundaries. Incidentally, my characterization of the EBM movement in terms of a resurgence of empiricism against rationalism is not new. In his brief survey of medical history, Warren Newton concludes that:

“At its founding, modern medicine represented a triumphal return of the rationalists. Their insistence on understanding the mechanisms of disease has been architectonic for modern clinical medicine. For all its rhetoric of novelty, Evidence Based Medicine represents a counter-revolution [toward] traditional empiricism, draped in modern clothes of statistics and multi-variate analysis. The tension between rationalism and empiricism abides and finds its expression in a wide variety of clinical questions.”

[5]

After summarizing the tension between rationalism and empiricism in the history of medicine, I consider the rise of the EBM movement in light of this history and as a movement that is prone to privilege the empirical strain over the rationalist strain. Finally, I speculate as to how formal training in philosophy and medical history might have furnished medical students and professionals with helpful insights for considering the role of expert advice in education and in evidence-based clinical epidemiology, a field in which rationalism and empiricism must work *in tandem*.

## 2. A Brief History of Medical Epistemology

A great deal of the history of philosophy can be viewed as a contest between those who emphasize deductive reason in the quest for knowledge (the “rationalists”) and those who emphasize sensory experience (the “empiricists”). As intellectual endeavors, medicine and biomedical research are no different. Many of the specific problems about knowledge in these fields are extensions of problems concerning human knowledge in general.

How do we know what we know?
What qualifies as knowledge in medicine or in the wider biological sciences?
How should physicians go about discovering new therapies?

These older, more foundational questions anticipate their modern counterparts, such as “what kinds of studies provide sufficient justification for choosing a particular therapy?” Presumably, a knowledge of epistemology can help the medical professional navigate these kinds of questions today, but crowded medical curricula leave little room for medical educators to accommodate significant study in the history of philosophy or the history of medicine. In addition, why should medical educators break their backs trying to do so?

In 1995, Rosenberg and Donald defined EBM as “the process of finding, appraising, and using contemporaneous research findings as the basis for medical decisions” [6] an apparently uncontentious goal. More commonly cited is the definition offered one year later by Sackett and colleagues: “the conscientious, explicit, and judicious use of current best evidence in making decisions about the care of individual patients [...] integrating individual clinical expertise with the best available external clinical evidence from systematic research” [7]. One wonders who could seriously oppose such a practice! By 1999, the zeitgeist was such that alternatives to EBM were the subject of frank parody in academic medical journals [8]. Moreover, the history of medicine is fraught with instances in which empirical testing—the clarion call of the EBM movement—has led to the correction of egregious errors on the part of more deductive or rationalistic practitioners.

In 1543, Belgian anatomist Andreas Vesalius published his seminal textbook *De Humani Corporis Fabrica* (“On the Fabric of the Human Body”), in which he recommended that every student of medicine practice anatomic dissection personally. Only by observing the human anatomy for themselves could students gain an accurate understanding of the body and—in contrast to generations of physicians before him—avoid the mistaken beliefs that followed from the uncritical acceptance of moribund theories from ancient authorities.

Even more infamous is the tragic account of Ignaz Semmelweis, who, backed by a preponderance of clinical data, recommended the practice of hand washing to medical professionals as a way of improving outcomes on the obstetrics wards in 1847 [9]. Despite empirical evidence that washing one’s hands between deliveries reduced mortality to below 1%, Semmelweis could produce no theoretical explanation or hypothetical mechanism with which to explain his findings. Being in conflict with the scientific opinion of the day, those findings were largely rejected by the medical community, and Semmelweis would only be vindicated posthumously with the emergence of germ theory—a theoretical model that placed the clinical data regarding hand washing and disease into an intelligible context.

Such epistemic tension in the medical profession can be traced back at least as far as the Greek physician Galen, who lived and worked throughout the Roman Empire during the 2nd century CE. His prodigious corpus became a major source for medical knowledge before his time and included works on clinical and scientific subjects such as anatomy, surgery, physiology, pathology, neurology, and pharmacology, as well as those that we would today consider philosophic in nature: logic and epistemology. (Galen authored a treatise titled “That the Best Physician is also a Philosopher”, for a detailed treatment of Galen and his thought, see [10].)

In the latter case, Galen was particularly interested in the debates between the medical sects of his day who were divided roughly along epistemological lines: the Empiricists, the Rationalists (or “Dogmatists,” according to Galen), and the Methodists, so named for their insistence on following a simple, algorithmic method for treating patients. (The Methodists I will not discuss at length, except to say that Galen found nothing commendable in their practices. Briefly, their theory consisted of a philosophical atomism like that of Democritus and Epicurus applied to human physiology. Their method amounted to facilitating equilibrium between the atoms of the body through good hygiene, diet, and the rare use of drugs. The Methodists were fond of claiming that an aspiring physician could learn everything there is to know about the craft in a few months’ time. This oversimplification irritated Galen, but his wholesale disregard of their work ignores several real accomplishments. For a review of Methodism and its founder Asclepiades, see [11] (pp. 3–7).)

According to Galen’s *On the Sects for Beginners*, medical practice in 2nd-century CE Rome was dominated by these three schools of thought [12] (pp. 3–11ff). The Empiricists believed that medical knowledge was gained by sensory experience, and to that end, they trained their pupils in the art of observation. No clinician could seriously disparage this approach, but the practitioners of this camp took it to the extreme. By and large, the Empiricists refused to speculate on the causes of disease or the mechanisms by which successful therapies acted. This refusal to engage in speculation ultimately resulted in a medical practice that was governed by correlation and association but was devoid of any scientific theory. With no models upon which to generate hypotheses, the Empiricists apparently relied on luck or “incidental” discoveries to generate new ideas for potential therapies. One baffling consequence of the empiricists’ method is that they typically had no interest in the human anatomy that was not visible to the naked eye [12] (p. xxvi).

The Rationalists, on the other hand, believed that theory ought to guide medical practice. Unlike the Empiricists, they were far less extreme in their commitment. Above all, the physicians of this sect sought a mechanistic cause for human ails. Armed with a causal understanding of pathophysiology, the Rationalists could thereby formulate principles by which to guide their practice.

The downside to this approach is that frequently their theories were wrong. When the Rationalists decided to treat patients according to a false understandings of human physiology and pathology, the consequences were at best ineffective, and at worst, disastrous. Even in the presence of valid reasoning, conclusions arrived at by deduction from prior theories are only as sound as the premises that those theories entail. Moreover, many such theories became so ingrained in the culture and practice of medicine that no one thought of challenging the traditional models by empirical observation or experimentation for centuries. Even in the age of modern medical science, the problem persists; it is succinctly given form by philosopher of science Holly Andersen, who explains: “a fundamental issue with respect to [mechanism-based reasoning] and EBM is that there is a discrepancy between knowledge of the causal mechanisms responsible for many functions, and knowledge about what will happen under intervention on those mechanisms” [13].

An ideal example of this rationalism-gone-wrong is the celebrated, and patently false, humoral theory of disease. Originating with Hippocrates ca. 400 BCE, the humoral theory persisted in some form well into the 19th century. (Published in 1851, Herman Melville’s classic novel Moby Dick begins: “…Some years ago—never mind how long precisely—having little or no money in my purse, and nothing particular to interest me on shore, I thought I would sail about a little and see the watery part of the world. It is a way I have of driving off the spleen and regulating the circulation.” The connection between Ishmael’s melancholic temperament and his spleen originates in the humoral theory of medicine. Black bile (or melancholy) was secreted by the spleen.) In this context, the word “humoral” comes from the word “humor”, meaning fluid. The human body was believed to play host to four such humors: black bile (also known as *melancholy*), red or yellow bile (*choler*), phlegm, and blood. In most cases, disease was the result of an imbalance between these four fluids. Each humor corresponded to a particular psychological experience, respectively: the melancholic, the choleric, the phlegmatic, and the sanguine. Each was also in possession of its own peculiar characteristics. For example, black bile was cold and dry, while yellow bile was warm dry. Phlegm was cold and wet while blood was warm and wet. Keeping these humors in equilibrium was one of the chief goals of medicine. A patient presenting with a melancholic illness, for example, would be suspected of excess black bile. The remedy for a preponderance of cold was therefore warmth, or drugs that promoted warmth.

## 3. Galen’s Synthesis

When one considers the humoral theory from the present day, it is difficult to see how the Rationalists of Galen’s day had any advantage over and against the Empiricists. In the face of such fanciful and speculative models of human physiology, the Empiricists seem to at least exhibit some restraint; instead of over-interpretation, they maintained an honest agnosticism.

Their *modus operandi* seems all the more responsible when one considers that, in practice, there was usually no difference in the prescription offered by practitioners of the different methods. The difference was found only in the method by which a remedy was found and how its use was justified. Galen observes that:

“…generally, the Dogmatists and the Empiricists draw on the same medicines for the same affections. […] In the case of the same manifest bodily symptoms, the dogmatists derive from them an indication of the cause, and, on the basis of the cause, they find a treatment, whereas the empiricists are reminded by them of what they have observed often to happen in the same way.”

[12] (p. 7)

Seeing as the prescribed treatments are going to be the same either way, why then would anyone prefer to indulge the speculative theories of the rationalists?

The answer lies in the power of mechanism-based reasoning to explain causation in ways that correlative studies cannot, and—importantly, for the progress of medical science—in their ability to offer warranted grounds for prediction in future studies. In contrast, the findings derived from empirical, clinical trials are frequently non-causal, and they make no claims about being so. Therefore they cannot be properly used to infer causal relations or generate new hypotheses for clinical intervention.

Better grounding in philosophy could have made this readily apparent to the medical community. To the epistemologically literate, any version of EBM that relies solely on the synthesis of findings from clinical trials very nearly runs the risk of repeating the mistake of logical positivism in the 20th-century—becoming self-defeating on its own terms. By privileging empiricism over rationalism, it becomes guilty of a paradox. Unqualified empiricism cannot, on its own terms, prove itself to be the preferred means of acquiring knowledge. What controlled study could be designed to demonstrate such a claim?

Granted, from a 21st-century perspective, it is easy to ridicule the erroneous theories of the Rationalist camp. The unwillingness of rationalistic practitioners to challenge ancient authorities or revisit old models deserves criticism. However, the rationalists of Galen’s era may have actually held the favored position for long-term progress in the medical sciences. Even though many of their theories were wrong, they at least had the epistemic infrastructure to gain real insight into the causal nature of human disease.

This possibility for progress on the part of the rationalists leaves one wondering where the empiricists would have gotten their ideas for new therapies. Intuitive hunches? Blind luck? Galen suggests that they relied rather on “transitions to the similar”—trying similar therapies for apparently similar disease presentations [14] (p. 5ff). Regardless, an exclusive appeal to empirical methods leaves one with the impression that medical practice was “flying blind”.

Part of Galen’s genius lay in his ability to synthesize both empirical and rationalistic methods into an epistemologically balanced approach, the kind of approach used in the scientific method in modernity. Nearly 1400 years before the birth of Francis Bacon, the notional founder of the scientific method, Galen describes the value of experimental science in which theoretical models are tested and refined empirically such that they can be treated axiomatically with greater faith. Evidencing his intuition for this kind of synthesis, Galen wonders why no one else “had ever taken the trouble to make a section themselves, or put a ligature around parts in the living animal in order to learn which function is injured” [15] (p. 70).

For an example of this, we might consider Galen’s famous experiments on the nervous system, wherein he systematically lesioned sections of the nerves and spinal cord to determine their effects. This in itself was a departure from both of the aforementioned schools. It was unlike the Dogmatists to the extent that it first employed experimentation prior to the formation of a theory. However, it also departed from the Empiricists to the extent that, firstly, it bothered to look “behind the scenes” at the internal workings of the body; and secondly, it sought to use observation in order to form a clearer mechanistic understanding.

During the vivisection of a squealing pig, Galen is further recorded to have accidentally severed the animal’s recurrent laryngeal nerve while searching for the vagus nerve [16]. Although it continued to struggle, the pig immediately stopped squealing, its powers of vocalization impaired. Speculating that homologous nerves might govern the same faculty in other animals, Galen validated his hypothesis by identifying recurrent laryngeal nerves in dozens of other animals, even going so far as to predict the risk of iatrogenic hoarseness or muteness for those who underwent the surgical removal of goiters.

Obviously, Galen’s attempt to synthesize the rationalistic and empirical strains in medical practice does not represent a wholly adequate scientific method; he still espoused and propagated many erroneous theories. However, Galen’s synthetic genius can be found in his use of empiricism within the confines of systematic experimentation. He used his observations to generate a mechanistic theory and, ultimately, to predict clinical outcomes based on a model (granting that these predictions would themselves be subject to further scrutiny by experimentation). Compared to the methods of his predecessors, Galen’s combination of rationalism and empiricism led to a deeper understanding of nervous injury. Moreover, his approach at least *anticipates* the scientific method, a method in which deduction (the primary method of the rationalists) and induction (the method of the empiricist) are both essential. To Galen, the best physician would be both a rationalist *and* an empiricist. He would use the power of observation to test his understanding of the causes of things, and thereby be able to make predictions.

## 4. Problems with Empiricism in EBM

As we approach the 30-year mark since EBM emerged to revolutionize the teaching and practice of clinical medicine, a vast literature of criticism has emerged to refine and, on occasion, reorient the movement. Backed by a passionate and energetic community, the accomplishments of EBM are many and real. However, the acceptance of EBM in the absence of a broader understanding of epistemology and medical history may have tarnished the movement’s legacy.

From its inception, critics asked whether the right kind of evidence would even be available in most cases. Would findings from homogeneous, correlative studies translate into decisions about real patients, many of whom are fraught with comorbidities? Would most patients even conform to “textbook” manifestations of the disease? According to research performed by the U.S. National Center for Health Statistics in 2012, 21% of adults between the ages of 45 and 64 years old were diagnosed with two or more chronic conditions. For those aged 65 years or older, this number grew to 45.3% [17]. This kind of multimorbidity frequently means that the highly controlled conditions contrived by a randomized trial do not represent the peculiar circumstances of a particular patient. Moreover, comorbidities introduce the possibility of complex interactions and pathophysiological mechanisms that ought to warrant serious consideration in spite of guidelines—a gesture toward rationalism and mechanism-based reasoning.

Granted that the responsible practice of EBM requires the practitioner to search the relevant literature and identify the best available evidence for a particular patient with particular characteristics, criticisms of EBM that are levied on homogeneous clinical trials may run the risk of attacking a straw man. However, Timmerman rightly notes that standardization, standards, and the search for “best practices” has long been one of the ways in which EBM has sought to address the problem of variation in clinical practice.

Others have worried that an overemphasis on empirical findings on the part of EBM would derogate research in the basic and mechanistic sciences in favor of large clinical studies, a move that could forestall progress in biomedical knowledge [4]. More simply, many physicians felt that EBM neither accounted for nor valued the knowledge that comes from clinical experience, nor the value of judgment in cases where an individual patient’s peculiar context or idiosyncrasies come into play.

Other publications to date have outlined the problems facing EBM [18]. The points made by these authors will not be elaborated here except to briefly illustrate where a privileging of empiricism over rationalism (or correlation over mechanism-based reasoning) has resulted in unintended consequences for clinical practice. The commentary regarding them is my own.

One problem is the sheer volume of evidence that an empirical model of practice would require clinicians to interact with on a regular basis. Citing a 2005 study in which 18 patients were admitted over a 24-h time period, Allen and Harkins found 3679 pages of evidence-backed guidelines relevant to those patients’ immediately diagnosable conditions [19]. At 122 h of reading for 18 patients in 24 h (approximately 7 h of reading per patient per day), any attempt on the part of a physician to “keep up with the literature” on every diagnosis would render the art of medicine literally un-practicable. In the context of competing epistemologies, the problem can be seen as one of too much empirical evidence and not enough reasoning based on disease knowledge and clinical experience. Intuition, pattern recognition, and deduction from basic principles are all rationalistic methods that can—and should—pare down the enormous amount of time spent consulting evidence-based studies. The alternative is a never-ending appeal to an insurmountable backlog of literature.

Another problem lies in the fact that large, correlative studies may reveal statistically significant findings and be methodologically sound while nonetheless remaining clinically trivial. No one would dispute the dramatic change in patient management brought about by the evidence that first-line “triple therapy” regimens for *H. pylori* infection are effective in the treatment of peptic ulcer disease. However, the reality is that few studies result in such drastic or standard changes to patient care. Rather, the majority of studies suggest improvements only on the margin, and many of these await reproduction in other settings. Sample sizes are large but not representative; significant differences in risk are measured by single digit percentage points. Further work by methodologists and improved study design may help to ameliorate these problems.

Even in the absence of comorbidity, differences in a patient’s social or cultural circumstances may call for personal judgment over and against empirically validated guidelines. Consider, for instance, the case of a 64-year-old man with urinary symptoms and a high risk of prostate cancer who is put on finasteride in keeping with the American Urological Association’s guidelines for prostate hyperplasia, but who, as a result, develops gynecomastia and consequently suffers social anxiety and depression. Or consider the diabetic 71-year-old woman with elevated LDL levels for whom the American College of Cardiology guidelines recommend statin treatment, but whose resulting muscle pains prevent her from exercising, and so her bone density measures fall precipitously and she succumbs to frailty. For a simpler example, consider the patient who—in a competent and informed state—rejects one treatment in favor of another less supported by the experimental literature. Granted that many proponents of EBM make room for *a priori* ethical principles such as patient autonomy, how would the committed empiricist be able to accommodate the principle of patient autonomy at all?

Finally, the zealous search for empirical evidence by the medical academy has resulted in a great deal of research that is consistent with the principles of EBM but is conducted without any prior attention to plausibility rooted in scientific (mechanistic) theories. The result is a host of expensive studies into the efficacy of alternative or scientifically dubious therapies, including those that Gorski describes as being tantamount to “testing whether magic works” [20]. Combined with the inevitability that many large studies occasionally turn up statistically significant (if trivial) results, the unintended consequence is an epistemically charitable environment for snake oil salesmen. The solution, according to Gorski, is surprisingly Galenic in character: “what is needed is science-based medicine rather than evidence-based medicine” [20]. Taking a leaf from the book of Galen, leaders in academic medicine should synthesize both empirical and rationalistic methods into an epistemologically balanced approach, the kind of approach that is already exemplified by the scientific method.

## 5. Conclusions

Along with the other humanities, what would now be called philosophy has historically constituted an important part of the physician’s education. In the medieval university, candidates for the higher doctorates in Theology, Law, and Medicine studied the liberal arts at the baccalaureate and master’s level before moving on to more specialized professional education [21]. When Aristotle was re-introduced to Western Europe during the 12th century, university-trained physicians studied the *Ethics* and *Politics* alongside his more scientific works such as the *Physics*. At the same time, the Holy Roman Emperor Frederick II required in his “Constitutions of Melfi” that all aspiring physicians in his realm precede their study of Hippocrates and Galen with the study of the humanities, “mindful of the health of our citizens and of the damage and suffering due to the inexperience of physicians…” [22]. Such a broad and philosophically rigorous course of study contrasts starkly with the present condition of medical education, where most students have no experience outside of the life sciences beyond their undergraduate institution’s common core or general education requirements.

As identified previously, the medical community in the United States faces a considerable gap in the humane and philosophic competence of its practitioners. The consequences of this gap are felt in various ways (Interested readers might consider the value of humane study for medical student professionalism [23] and basic medical science research [24]), including the lack of nuance that medical education has displayed in addressing the issues previously described. Better training in philosophy might lead medical students and educators to acknowledge that empiricism is not—in an unqualified sense—superior to other instructional approaches on epistemic grounds. Rather, it is only part of the answer to a longstanding question dating back at least to ancient Rome: “What is medical knowledge, and how should we apply it?” Despite widespread bias in its favor, a positivistic system that privileges empiricism over rationalism or mechanistic reasoning is not by itself capable of arriving at medical knowledge without presupposing theories rooted in the basic sciences. As Galen understood, both approaches contribute something to the physician’s understanding of her craft, but each is extreme, simplistic, and inadequate in isolation. Significantly, Galen is not the only figure from medical history to effect a “Galenic synthesis” of this kind. William Harvey, the celebrated physiologist and so-called father of cardiology, also represents an attempt by a philosophically literate physician-scientist to unite the rationalistic and empirical tendencies in the medical practice of his day. Primarily an empiricist and a disciple of Aristotle, Harvey nonetheless affirmed the need for starting with mechanistic theories built on *a priori* knowledge [25].

As I have argued, the scientific method (and EBM, to the extent that it is a scientific enterprise) requires both observation and deduction from mechanistic theories if it is to be practiced effectively. However, even as late as 2011, the Oxford Centre for Evidence-Based Medicine lists mechanism-based reasoning among the least reliable forms of evidence, defining the practice as “an inference from mechanisms to claims that an intervention produces a patient-relevant outcome” [26]. Granted that any sound inference from a mechanistic theory will be limited by the correspondence of the theory in question to reality, the Centre for Evidence-Based Medicine would seemingly disparage the very notion of causation in medicine!

Even so, there are reasons to remain hopeful. One recent article describes an educational program called “R3” designed to promote scientific rigor, responsible research, and reproducibility in the medical sciences. Intended specifically for PhD students in the medical sciences and public health, R3 summarizes its goal as one of “[putting] the philosophy back into doctor of philosophy.” The pilot program, which includes required coursework in epistemology and ethics, is an encouraging example of philosophically informed training in the medical sciences [27]. Whether programs in clinical medicine decide to follow their lead remains to be seen.

As illustrated by the problems previously identified, an extreme allegiance to empiricism is not sufficient. The kind of medical knowledge gleaned through stark empiricism resists understanding until it can be fitted into a theoretical or mechanistic context. The chicness and common-sense appearance of EBM no doubt appealed to educators and administrators in the 1990s, but their implementation of the paradigm in the years since proved that they did not anticipate the vulnerabilities of EBM where it embraced empiricism out of proportion to rationalism. What might have helped is a knowledge of the history of philosophy, at least as it relates to medical epistemology.

As previously conceded, empiricism proves very useful when challenging false “concepts”. Many theoretical models are demonstrably wrong, and no doubt many more will be shown to be likewise. The humoral theory, however useful it may have been at one time for organizing clinical findings in an intelligible way, is one such concept, and experimental methods have since corrected it. Rather than committing to an unexamined empiricism, however, EBM should take a leaf from Galen, who sought to synthesize the best elements of the Empirical and Rationalist traditions. To do so is to join the company of another great epistemological unifier, Immanuel Kant, who famously asserted that “Concepts without Percepts are empty; Percepts without Concepts are blind” [28] (p. 50). To fail to understand EBM in its wider epistemic context is to privilege percepts over concepts, an overcorrection that is prone to other kinds of errors. In addition, a medical practice that is guided only by percepts is a medical practice that is blind.
